# Supplementary material for: Tropical fishes vanished after the operation of a nuclear power plant was suspended in the Sea of Japan
Source: PLoS One. 2020 May 6;15(5):e0232065. doi: 10.1371/journal.pone.0232065 (PMC7202591; doi:10.1371/journal.pone.0232065)
Supplement: Attachment — Submitted filename: responses.doc.docx [file pone.0232065.s002.docx]

Response to Reviewers

I wish to re-submit the manuscript titled **“Tropical fishes vanished after the operation of a**

**nuclear power plant was suspended in the Sea of Japan”** The manuscript ID is: Ref.

PONE-D-19-04526R2

Thank you for your e-mail on 2 October 2019 regarding the above mentioned manuscript. I asked

for statistical advice from Dr. Yutaka Osada who is a specialist of theoretical ecology and is

competent with statistics. His opinion was that constructing two-factor ANOVAs including

factors of time (years) and sites followed by post-hoc tests would guarantee the objectivity of the

study, while nested ANOVA dividing into two period will provide a rigid comparison between

before and after the event. He also advised that Tukey’s HSD test is conservative enough so that a

Bonferroni correction among multiple Tukey’s tests is not required. The statistics of the

manuscript is thus revised. The main result represented in Figure 2 is now based on two-factor

ANOVA followed by Tukey’s test comparing sites within years. Comparison of tropical fishes

between pre- and post suspension was conducted by the nested ANOVA as in the previous

manuscript. This way the description of the results in Figure 2 has been simplified and repetition

in Figure 3 has been avoided. Revisions have also been made according to the specific

suggestions provided by the editor.
