# Supplementary material for: Tropical fishes vanished after the operation of a nuclear power plant was suspended in the Sea of Japan
Source: PLoS One. 2020 May 6;15(5):e0232065. doi: 10.1371/journal.pone.0232065 (PMC7202591; doi:10.1371/journal.pone.0232065)
Supplement: Attachment — Submitted filename: Response_to_Reviewers.doc [file pone.0232065.s003.doc]

**Responses to Editor and Reviewer Comments**

**Academic Editor:**

While the statistical rigor of the manuscript has been improved, both reviewers have identified issues with the paper, mainly around the statistical analyses, that need to be rectified. Reviewer 1 has noted that the PERMANOVA design should be the same as the univariate design and also has provided a number of comments to clarify the text, which is often confusing. Reviewer 2, who has not seen the manuscript before, has identified more serious issues with the statistics, including that some of the analyses are unbalanced due to missing data and that it appears the author did not follow the advice of the statistical advisor they consulted and tests for differences between time periods (i.e. when Otomi was operating and when it wasn't) were not done. In addition, the reviewer has noted that where the data are unbalance mixed model analyses are preferable to fixed effect ANOVAs, although these are more complicated.

**Response:** Thank you for identifying the flaws in statistics. PERMANOVA has been conducted in the form of pre- and post-suspension comparison for each site as suggested by Reviewer 1. Generalized Linear Mixed Model (GLMM) has been conducted in the revised manuscript with a help of a statistician.

**Reviewer #1: General Comments:**

This manuscript describes changes in fish assemblages before and after the suspension of a nuclear power plant in the Sea of Japan. The article is generally well-written, however there are numerous issues regarding statistical analyses and interpretations. One specific issue that affects the overall concept of using the NPP as an indicator of future warming environment is that the temperature at NPP was only significantly different to reference sites in a single year prior to the suspension of NPP discharge (Fig 2a). I suspect this may relate to the lack of power from using only four temperature measurements at each site within each year, because the temps at NPP look considerably higher than the reference sites in Fig 2a. One way to avoid this would be to just present the temperature values, rather than statistically testing them, or pool temperature measurements before and after the suspension and make a single comparison for each site. I provide detailed comments below to assist a revision.
**Response:**

I thank the reviewer for this general comment. At the first sentence of the result, I presented the temperature values to show the drop of temperature with the suspension of NPP. GLMM analysis has also supported the abrupt temperature change in 2012. I found an error in applying Tukey’s test in my previous analyses. Since GLMM is suitable for the analysis for the general result presented in Fig 2, ANOVA for these data were deleted.

**Specific comments:**

Title: replace ‘vanish’ with more scientifically-appropriate word
**Response:**

I consider this suggestion very carefully. I could replace “vanished” with “disappeared”. However, I think that the abrupt disappearance of tropical species may be expressed better with the term “vanish”.

Line 33-42: Suggest moving this paragraph further down and opening the introduction with the information about climate change. The power plant is really just an opportunistic ‘method’ for investigating potential assemblage responses to increasing temperature sin the region.

**Response:**
Introduction has been changed to open with the general notion of global warming as is suggested by the reviewer.

Lines 51-54: Citations/references required to support these broad statements.
**Response:**
Following references have been cited as suggested:

1. Sortes CJB, Williams SL, Carlton JT. Marine range shifts and species introductions: comparative spread rates and community impact. Global Ecol Biogeogr 2010; 19: 303-316.

2. Brander KM. Global fish production and climate change. P Natl Acad Sci 2007; 104: 19709–19714.

Line 75: suggest adding, “... as a proxy for understanding the impact of future temperature increases in the region”. Otherwise we are back to the purpose being about thermal discharge impacts, rather than what the authors state further up in the introduction regarding thermal plants simply acting as a model system for understanding climate change effects.

**Response:**
Revision has been made according to the suggestion. This way general introduction starting with climate change is tightly connected with the study on thermal discharge impacts.

Lines 77-79: Figure 2a actually suggests there are few years where the temperature at the NPP site is significantly higher than the reference sites. In my opinion, the graph does depict a considerable difference in temperatures, and the standard errors are quite small, so I’m not sure why there are so few statistically-significant differences in temp. Perhaps values from different seasons

**Response:**
I found some errors in my previous Tukey’s test analyses. In the revised manuscript the focus is on the GLMM here. This way the temperature difference has become highly significant between pre- and post- suspension periods.

Line 119-120: what precisely was the decision based on – ease of sampling, depth, similar habitat etc?

**Response:**
The area was decided to maximize the detection of rare species in limited diving time. This has been clarified in the revised manuscript (L. 123-125) as follows:

The size of survey areas was decided to maximize detection of rare species within limited diving time based on previous studies conducted at the Nagahama and Otomi sites for other purposes [4, 18].

Line 124: how many surveys were conducted at each site in each year?

**Response:**
I conducted four surveys at each site in each year. This has been described further up in this section (L. 104).

Notes: Different depths – the NPP site is considerably shallower than either of the other two sites. Some discussion of this potentially confounding effect on comparisons between treatment and control sites is required.

**Response:**
Average depths were comparable between the Otomi and Nagahama site, but the depth of the latter was more variable. The potential confounding effect of such differences has been mentioned in the revised manuscript (L. 302-304) as follows:

The Otomi site was consistently shallow, whereas other two sites had more variable depths in the survey area; such differences also have potentially confounding effect on comparisons.

Line 133-134: Rephrase this description, specifically “the criteria of relative abundance in the southern species”. Suggest the authors consider COD as an indicator of average latitudinal distribution within the assemblage, which when compared through time, can be used to understand trends in ‘tropicalisation’ of an assemblage.

**Response:**
I thank the reviewer for notifying this ambiguity and redundancy. The logic in the previous description may have been circulatory. The description has been revised as “the criteria of spatiotemporal tropicalization” (L. 138-139).

Line 144-145: It is not possible to nest surveys within years – surveys represent the unit of replication, from what I can tell. The analysis does make sense if the authors nested years within periods, i.e. before and after suspension of the NPP.

**Response:**
I thank the reviewer for identifying this misuse of terms. The wording has been revised as suggested as follows (L. 152-153):

(i.e., survey years were nested in each period).

Lines 142-145: More detail regarding the statistical design is required here. Were three separate ANOVAS conducted, one for each category? This seems to be the case form the wording of the results. So three separate three-factor analyses (one for each category), with factors of year (nested in period), period and site?

**Response:**
I had conducted four separate two factor ANOVAs with factors of years and sites for the analysis in Fig. 2. I found that Tukey’s test was improper for this analysis. In the revised manuscript I conducted analyses with generalized linear mixed model (GLMM) to confirm that all response variables changed between the two periods (up to 2011 in temperature, species richness and center of distribution and up to 2012 in fish abundance). Then comparisons have been focused on the comparison between pre- and post-suspension period in the following sections. The statistical design has been detailed in the revised manuscript.

Line 149: what were the plotted data visually inspected for?

**Response:**
The above description has been deleted in the revised manuscript.

Lines 149-152: The PERMANOVA design should follow the same two-factor design used in the univariate comparisons. There is no (deductive) logic associated with plotting data, looking to see groupings (separations) and then constructing a statistical test to verify apparent visual differences, which is what the author appears to have done according to the description provided in this section.

**Response:**
The PERMANOVA design has been revised to compare two periods in each site according to the suggestion (L. 157-158).

Lines 155-156: Rephrase. The temperature actually appears to decrease linearly between 2009 and 2014. A substantial drop in temperature was also recorded during 2008, prior to the suspension of the NPP. Why?

**Response:**
The description of the temperature decrease has been revised to be based on facts referring raw data (L. 163-165). Relatively low temperature in 2008 may be due to heavy snow fall in this year.

Lines 163-165: I’m surprised by the low number of significant results, given the relatively small error indicated by the bars in Fig 2a. Perhaps Tukey’s HSD is too conservative here?
**Response:**
I found a misuse of Tukey’s test here, so that the suggested part has been deleted in the revised manuscript.

Line 164: significantly warmer than the reference sites I assume. Insert the clarification.
**Response:**
The description has been removed in the revised manuscript.

Lines 164-165: suggest rephrasing to, “…whereas no differences were found among sites in other years” for clarity.
**Response:**
This phrase has also been removed.

Line 178-182: also need to state here that no significant differences were found between Otomi and the other reference site.

**Response:**
This phrase has also been removed.

Lines 182-184: I recommend adding the results of the significant interaction term here in brackets, to support this statement regarding before/after. Interaction between sites and years is what you would expect if trends at one sites (hopefully NPP) differed to those at the other sites.
**Response:**
ANOVA for this part has been deleted.

Lines 193: Which model term (factor) is this p-value for? See previous comments regarding more detail required on statistical design, but if species categories have been included as a factor in the analysis, I would expect to first see an interaction term reported, specifically the interaction between category and period. This would indicate a possible difference in trends of tropical species between the two time periods, but not subtropical and temperate species.

**Response:**
In this section ANOVA was applied for each category of fishes, i.e., tropical, subtropical or temperate fishes, in each site separately. Therefore, p-value was only for the comparison between pre- and post- suspension periods of NPP. This has been clarified in the method and result descriptions (L. 149-153 and 213-220).

Lines 26-209: Again, no logical reason for structuring the analysis around the four groups seen in MDS. The statistical design should be decided a priori, similar to the univariate tests described for abundance and species richness.

**Response:**
This section has been revised so that comparisons were conducted between two periods in each site.

Line 217-225: Suggest quantifying ‘dominance’ in some way, perhaps by reporting percentage of total fish abundance for each of these species.

**Response:**
Percentages of total abundance in these dominant species have been reported in the revised manuscript as suggested by the reviewer (L. 243-254).

Lines 229-234: worth mentioning here how many days after the NPP suspension the surveys were completed

**Response:**
Tropical species were not found in any winter for the following 5 years after the survey. Then the NPP restarted operation in May 2017. I am still continuing the survey and found that tropical species are gradually settling. This will be another fish to fry after the publication of the present paper.

Reviewer #2: Reviewer Report for PONE-D-19-30294

Tropical fishes vanished after the operation of a nuclear power plant was suspended in the Sea of Japan PLOS ONE

Summary of Research:
The research examines a marine ecosystem that developed in the thermal outflow from a nuclear power station, and observed what happened when the artificial heat source was cut-off. These observations were compared with 2 other sites, one a coal fired power plant with smaller thermal output, and a control site with no artificial heat source.

Summary of impression:
My impression was of a unique study that entailed a great deal of detailed and well thought out work, with some strong findings. However, the analysis section is not optimal, and is incomplete. Presentation of findings should also be improved.
**Response:**
I thank the reviewer for evaluating this work. The analyses have been substantially improved in the revised manuscript according to the suggestions.

Major issues:
Statistical analyses are not optimal, to the point that some required treatment effects were not properly tested. Site*Year statistical models might be partly appropriate for Sezaki and Nagahama, but are not optimal for Otomi. The most appropriate models for Otomi need to test the 2 level time-period effects, which should also be applied to Sezaki and Nagahama to test if these sites differ in this respect to Otomi. I have more detailed suggestions below.
**Response:**
I agree with the reviewer that Site*Year model was not optimal for the goal of this study. Thus, the above analysis was abandoned and GLMM has been applied in the revised manuscript.

Minor issues:
Some detail is missing from the methods (P.7) that would otherwise clarify the areas that surveys were sampling at each site. Was each survey strip at each site selected at random, per survey instance, or was the one strip locations used at each site over all years? If transec strips are chosen at random at each site, what spatial area is being sampled to represent each site?.
The discussion could suggest bases for observed differences between Sezaki and Otomi, which might provide more perspective to the study. Otomi started in the 1970's and has high thermal output, Sezaki started in 2004 with smaller thermal output, Otomi had tropical fish species establish by ~30 years, and Sezaki didn't by 0-7 years.
**Response:**
Methods of the survey has been described in more detail in the revised manuscript (L 119-125). The survey strip is not totally random, yet not totally fixed. This way the detection of relatively rare species was maximized in limited diving time.

For the detailed comparison among survey sites, I think there are some limitations. No data was available concerning what occurred in Otomi during 1970’s, and Sezaki differed from Otomi both in the thermal output and geographic location. Therefore, in this manuscript, I would like to refrain deeper discussions to compare the sites other than the suspension of NPP.

Other comments:

Figures and Tables-

⦁ Figures would be improved by placing 95% confidence intervals around all means. Currently Fig 2 uses standard errors of means, and Fig 3 uses standard deviations, the former is better for purpose than the latter, but both are inferior to confidence intervals for allowing visual evaluation of results. One wouldn't need to conduct Tukey tests if 95% confidence intervals were used to highlight which treatment levels differed most, in situations where ANOVA's showed treatment levels differed significantly.
**Response:**
Error bars in the previous Fig 3 have been replaced by standard errors as suggested. I was not sure how to draw 95% confidence limit with the graphic software I use (Kaleida Graph).

⦁ Tables. Table S2 reports 2-factor ANOVAs (site x year), which don't address all the needs of the study. The tests for differences between time periods within sites were not fitted and reported. By "time periods" I mean when Otomi was operating, and when it wasn't. The author did not adopt the advice of his own advisor, to fit these terms in his ANOVA models.

**Response:**
ANOVA corresponding to Fig 2 was not efficient. Therefore, GLMM has been applied here to confirm the difference between two periods. Time period effect on each variable as well as interaction have been reported based on the GLMM results in the new Table 1 in the revised manuscript. Then the fish assemblages in two periods were further compared with the new Fig 4. I hope this makes more sense.

⦁ The plot of fish assemblages under multidimensional scaling (Fig 4) could be discussed in more detail. What variables were the X and Y axis dimensions mostly reflecting? The X axis dimension separates Nagahama from Sezaki and Otomi, and the Y axis doesn't. Related to this perhaps, is why may Otomi "off" attach to Sezaki, and is then furtherest from "Nagahama"?
**Response:**
I thank the reviewer for notifying these interesting aspects. In my understanding it is not possible to detect what factor is contributing to each axis in nMDS. The reason Otomi was off attached to Sezaki and far from Nagahama in the nMDS may be that Nagahama is located in highly protected inner bay, while location of other two sites are relatively exposed, as is seen in Fig 1. This is included in the revised discussion as follows (L 298-300):

Common character of relatively exposed location in the Sezaki and the Otomi sites may have contributed to the closer characteristics of fish assemblages represented in the nMDS (Fig 5).

Methods- Are generally well detailed.

Study Design- Was the best that could be done, for the study objectives. Being an observational study, it is difficult to fully control differences between sites.

Results and data- The results generally seemed to support the study conclusions. I didn't fully agree with the author, regarding his findings from Fig. 3 (P 10, lines 193-197), but I may have been misled by the SD bars in this figure.

**Response:**
SD bars have been replaced by SE bars. There were some flaws in the plotting of SD in the previous version of the manuscript. They are all rectified. SE bars are now consistent with p-values in the revised new Fig 4.

I couldn't find any accessible temperature data, to accompany the observations on fish communities (latter provided).

**Response:**
Temperature data of all the survey sites and dates have been included in the 5^th^ line of the S1 Table.

References- References should be given for statistical tests, as well as an appropriate reference for the R-software.
**Response:**
Five references are given for R-software itself and install packages in the revised manuscript (References 21-25).

Statistics-

Analysis of the data largely uses ANOVA, with output from the ANOVAs used to generate errors for means of interest, and to underlie Tukey's HSD tests. The ANOVA's that are reported are 2 factor Site*Year tests (Table S2). ANOVAs involving some form of nesting are briefly alluded to, but any findings from nested analyses were not reported (line 144, "surveys" under "site*years" were not analysed as nested effects). Year effects can be nested under time-periods, but these analyses were not conducted for the current manuscript. In these circumstances, reference to "nesting" should be removed from the manuscript.

The ANOVA's reported in the paper, don't optimally meet the needs of the study at Otomi, in that the factorial models site*year are of lesser use than testing time period effects (the years when NPP Otomi is operating and when it isn't). The site*year ANOVA is useful for generating SE's of means for within years between site effects (Fig. 2), but even for temperature, only 1 within year between site comparisons was found to be significant. The "time period" within site effects are better illustrated in Fig. 3, but ANOVA's to test these effects were presumably not conducted. It is unclear how the SD's for Fig. 3 were generated.

**Response:**
I thank these comments by the reviewer. Tukey’s test in Fig 2 did not make much sense so I deleted them. I did conduct ANOVA for the analysis in the previous Fig 3, the results of which have been more detailed in the revised manuscript.

The experimental design for analyses with time period effects is not balanced for site, as Sezaki data is missing for years 2015-2017. As the Sezaki CPP wasn't operating in the winter of 2004, that years Sezaki data shouldn't be included in any time-period analysis either. Also, Otomi was switched off on Feb 20th 2012, in the middle of transec surveys, so subsequent 2012 data at this site should be dropped from the analysis, and depending how quickly a nuclear power plant can be shut down, and when this process started (if well before Feb 20th??), perhaps the Otomi 2012 data should be dropped entirely?. Of interest, was Otomi operating at the time of the winter surveys in 2008?, from Figure 2 it doesn't look as if it was. Where there is unbalanced data, mixed model analyses are much preferable to straight fixed effect ANOVA's.

Inspection of Fig. 2 suggests models in which to fit "time-period" effects for separate analyses for fish abundance, species richness, and temperature. Models might begin with a single "spline" across years and sites as a fixed effect, followed by "sites/time periods" as further fixed effects, and year as a random effect. Fitting fixed and random effects in a statistical model, complicates deterministic prediction of confidence intervals of means (for presentation in figures). Confidence intervals can then be derived stochastically, using the "sim" function in R, once a satisfactory mixed model is fitted. Benefits of such an approach are ability to test "time period" effects, have more powerful tests in the process than just "site*year", and to substitute 95% confidence intervals for presentation of results in figures, instead of using Tukey tests to identify which treatment levels are differing.

The models fitting time-period effects need to be analysed by an experienced statistician.

**Response:**
I thank the reviewer for providing the idea of analysis. I consulted to a statistician, and according to his advice, GLMMs have been conducted in the revised manuscript as is represented in new Fig 3 and Table 1. This way the data in the year 2012 have been made use of.
