# Supplementary material for: Tropical fishes vanished after the operation of a nuclear power plant was suspended in the Sea of Japan
Source: PLoS One. 2020 May 6;15(5):e0232065. doi: 10.1371/journal.pone.0232065 (PMC7202591; doi:10.1371/journal.pone.0232065)
Supplement: Attachment — Submitted filename: Example code for PLOS author.doc [file pone.0232065.s004.doc]

```
R-code used….

To generate year means for temperature data, and then plot, with lines linking adjacent years, for each site


rm(list=ls(all=TRUE))

setwd("G:/FisheriesAndQuantitative/……….")
library(lme4)
library(arm)

temp.dat <- read.csv("S1_Temperature_Japan.csv")
temp.dat$Replicate <- as.character(temp.dat$Replicate)
temp.dat$Year <- as.character(temp.dat$Year)
temp.dat$Code <- as.character(temp.dat$Code)

  yrN.grid <- yrO.grid <- seq(2004,2017,1)
  yrS.grid <- seq(2004,2014,1)
  y.grid <- list(yrO.grid,yrS.grid,yrN.grid)

tsite=c("Otomi","Sezaki","Nagahama")
tcol=c("red","green","blue")

pdf(file="Pos_Consig_for_ViroidsD.pdf",paper = "a4",pointsize = 10)
plot(temp.dat$Year,temp.dat$Temp, ylab ="Temperature (oC)", xlab = "Year" )

# Use this code to directly fit year-means for data
for(j in 1:3) {
  temp.datR <- temp.dat[(temp.dat$Site == tsite[j]),]
  fit.yr <- lm(Temp ~ Code,data=temp.datR)                  # not an intended formal analysis
  nYr <- length(unique(temp.datR$Year))
  tyR <- as.numeric(fit.yr[[1]][1] + c(0,fit.yr[[1]][-1]) )
 lines(unique(temp.datR$Year), tyR, col=tcol[j],  lwd=2 )
 points(temp.datR$Year,temp.datR$Temp, col=tcol[j] )
  rm(fit.yr,nYr,temp.datR )
}

legend("topright", c("Otomi","Sezaki","Nagahama"),
       lty=rep(1,3),lwd=c(2,2,2),col=tcol,bty="n",cex=1 )
dev.off()


# resultant plot from above code, as immediately below.  Clearly shows consistency of site rankings, despite variation in yearly temperatures.  Also Otomi rank reversed after 2012….
```

```
Separately analyse temperature data within 2 Otomi operation states, using mixed models, and generating 95% confidence intervals for within state site means

#################################################################################
# having established how best to analyse temperature data, now run mixed models
#       within time periods, and generate confidence intervals via simulation of fits.

rm(list=ls(all=TRUE))

Temp.dat <- temp.dat
Time.dat1 <- Temp.dat[(Temp.dat$TPeriod == 1),]

fit.T1 <- lmer(Temp ~ -1 + Site + (1 | Year/Replicate ), REML=T, data = Time.dat1 )
plot(fit.T1)               # check model of fit, fitted versus residuals
summary(fit.T1)            # summary of the fit

# determine p for fitting "site", Chi-square test; Otomi “on”
fit.T3 <- lmer(Temp ~ 1 + Site + (1 | Year/Replicate ), data = Time.dat1, REML=F )
fit.T4 <- lmer(Temp ~  1 + (1 | Year/Replicate ), data = Time.dat1, REML=F  )  anova(fit.T4,fit.T3)


# simulate the model to get CI's for fixed effects (nb. random effects sum to zero)
n.sims <- 10000                   # to simulate ten thousand times
fit.sim <- sim(fit.T1,n.sims)     # generate the simulations given the model fit
predFE.lmer4 <- fit.sim@fixef     # extract estimated fixed effects for each simulation
apply(predFE.lmer4,2,function(x) quantile(x,c(0.025,0.5,0.975)))  # get quantiles ex 10k sims


#################################################################################
# now repeat process for time period 2; Otomi off
rm(list=ls(all=TRUE))
Temp.dat <- read.csv("S1_Temperature_Japan.csv")
Time.dat2 <- Temp.dat[(Temp.dat$TPeriod == 2),]
fit.T2 <- lmer(Temp ~ -1 + Site + (1 | Year/Replicate ) , REML=T  , data = Time.dat2 )

plot(fit.T2)
summary(fit.T2)

# p for fitting "site", ", Chi-square test; Otomi “off”
fit.T3 <- lmer(Temp ~ 1 + Site + (1 | Year/Replicate ), data = Time.dat2, REML=F  )
fit.T4 <- lmer(Temp ~  1 + (1 | Year/Replicate ), data = Time.dat2, REML=F  )  anova(fit.T4,fit.T3)


# for each fitted model, simulate the model to get CI's for fixed effects (random effects sum to zero)
n.sims <- 10000                   # to simulate ten thousand times
fit.sim <- sim(fit.T2,n.sims)     # generate the simulations given the model
predFE.lmer4 <- fit.sim@fixef
apply(predFE.lmer4,2,function(x) quantile(x,c(0.025,0.5,0.975)))


#################################################################################

Resultant Confidence intervals are placed in a csv file, to be read by another program to plot the site means with 95% confidence intervals


R-Code to generate plots of site temperature means with confidence intervals generated above


Confidence intervals in “Marine_Site_temps.csv” file, for plotting of confidence intervals

NPP.stat,Site, LTB,  MTR,  UTB
on,   Nagahama,11.65,12.21,12.76
on,   Sezaki,  11.08,11.63,12.18
on,   Otomi,   13.06,13.61,14.17
off,  Nagahama,11.15,11.79,12.44
off,  Sezaki,  10.58,11.36,12.14
off,  Otomi,    9.96,10.6, 11.23


# code to plot fixed effects and 95% confidence interval for site, using site Temperature data

rm(list=ls(all=TRUE))
setwd("H:/My Documents/……")
dat.T <- read.csv("Marine_Site_temps.csv")  # file immediately above

XD <- c(0.25,0,-0.25)
SC <- c("blue","green","red")

pdf(file="Marine_Site_temps.pdf",
    paper="a4", pointsize=10 )
par(mfrow=c(1, 1))

plot(1:6,dat.T$UTB, pch=" ", xlim=c(0.5,6.5), ylim=c(9,15), ylab="Temperature",
     xlab="Otomi NPP operating periods (2004 - 2017)", xaxt="n" )
  text(2,9.2,"Otomi, ON (2004-2012)")
  text(5,9.2,"Otomi, OFF (2013-2017)")
  lines(c(1,3),c(9.05,9.05),lty=1 ); lines(c(4,6),c(9.05,9.05),lty=1 )
  lines(c(3.5,3.5),c(9,15),lty=3)

dat.Ton <- dat.T[(dat.T$NPP.stat == "on"),]
for(d in 1:3){
  points(d+XD[d],dat.Ton[d,4],col=SC[d],pch=16,cex=1.5)
  arrows(d+XD[d],dat.Ton[d,4],d+XD[d],dat.Ton[d,3],
         length=0.15,angle=90,code=2,col=SC[d],lwd=2)
  arrows(d+XD[d],dat.Ton[d,4],d+XD[d],dat.Ton[d,5],
         length=0.15,angle=90,code=2,col=SC[d],lwd=2)
                }

dat.Ton <- dat.T[(dat.T$NPP.stat == "off"),]
for(d in 1:3){
  points(d+3+XD[d],dat.Ton[d,4],col=SC[d],pch=16,cex=1.5)
  arrows(d+3+XD[d],dat.Ton[d,4],d+3+XD[d],dat.Ton[d,3],
         length=0.1,angle=90,code=2,col=SC[d],lwd=2)
  arrows(d+3+XD[d],dat.Ton[d,4],d+3+XD[d],dat.Ton[d,5],
         length=0.1,angle=90,code=2,col=SC[d],lwd=2)
}

legend("topright", c("Nagahama","Sezaki","Otomi"),
       lty=rep(1,3),lwd=c(2,2,2),col=SC,bty="n",cex=1 )
dev.off()


Plot on next page


file="Marine_Site_temps.pdf",  as generated immediately above

Otomi on, Nagahama significantly colder than Otomi, differ at ~ p=0.01 (on basis of 95% CIs)
Otomi off, Nagahama significantly warmer than Otomi, ~ p=0.05 (on basis of 95% CIs)
```

Having run the above code, I got somewhat different parameter estimates to your results.

I am not sure what is the cause of this, but doesn’t really matter at this stage, I just intend to illustrate getting 95% confidence intervals and plotting them for a Fig. 4 type display and interpretation.

The mixed model authorities suggest putting more faith in the confidence intervals, than Chi-square, F and t statistics, though more recent R-software may have improved things somewhat ????.

Good luck.
