# Supplementary material for: Tropical fishes vanished after the operation of a nuclear power plant was suspended in the Sea of Japan
Source: PLoS One. 2020 May 6;15(5):e0232065. doi: 10.1371/journal.pone.0232065 (PMC7202591; doi:10.1371/journal.pone.0232065)
Supplement: Attachment — Submitted filename: PLoS editorial comment_NPP.docx [file pone.0232065.s005.docx]

**PLoS editorial comment_NPP**

Line 33: Should be ‘are major concerns’

Line 45: Should be ‘This is much faster’

Line 46: I would say ‘global average’

Line 53: Should be ‘than the present level, respectively, under…..’

Line 54: Don’t introduce the abbreviation IPCC here as it is not used again

Line 69: Write as ‘where the visual censuses’

Line 140: Don’t introduce the abbreviation GLMMs as it is only used once more (PLoS formatting is that abbreviations need to be used at least 3 times)

Lines 142-143: This is confusing as written so please clarify ‘here all…..’ Not sure what the author is saying. All the combinations were separated? Not sure what that means.

Line 177: Should be ‘the two periods’

Line 190: Should be ‘models’

Line 192: Use the established abbreviation ‘COD’

Line 202: Confusing as written ‘between up to’? Would be better to say between this year and this year so the period is clear.

Line 246: Delete the space between 12.9 and % (so write as 12.9%)

Line 248: Delete the space between 23.0 and % and insert ‘,respectively’

Line 251: Delete the space between 9.3 and % and insert ‘,respectively’

Line 254: Delete the space between 5.8 and % and insert ‘,respectively’

Line 289: Should be ‘assemblages’

Line 290: Write as ‘the Sezaki site actually had significantly higher CODs, representing more cold water’

Line 291: ‘site’ should not be capitalised

Line 292: Write as ‘vagrants’

Line 295: Should be ‘as a potential factor’

Line 298: Confusing as written. Maybe say ‘Common habitat characteristics of the relatively exposed locations of the Sezaki and Otomi sites’ or common geographical characteristics? Something like that.

Line 302: Should be ‘whereas the other two sites’

Line 307: Should be ‘southern areas’
