# Supplementary material for: Tropical fishes vanished after the operation of a nuclear power plant was suspended in the Sea of Japan
Source: PLoS One. 2020 May 6;15(5):e0232065. doi: 10.1371/journal.pone.0232065 (PMC7202591; doi:10.1371/journal.pone.0232065)
Supplement: Attachment — Submitted filename: Response.docx [file pone.0232065.s006.docx]

**Responses to Editor and Reviewer Comments**

**Academic Editor:**
I appreciate the huge amount of work the author has done to improve this manuscript. And I think it certainly has improved. As noted by Reviewer 2, I think this decision is halfway between major and minor revision required. I think the text has greatly improved and the use of GLMMs in the paper has improved the analyses. I think the problem with the paper is that is it not entirely clear what exactly has been done and the presentation of the results could be better. So I think the data and the story are there, it is just making it very clear that is important. As currently written it is quite confusing.

Reviewer 2 in particular has provided very detailed comments and suggestions to improve the analyses and the explanation of what was done. They have also provided example code to improve the presentation of the results. I know this paper has been reviewed quite a few times already, but I think it is getting very close to where it needs to be. Again, the author has at least attempted to undertake all the comments and suggestions provided so that is appreciated. I encourage the author to consider all the comments provided when making their revisions.

**Response:** Thank you for your help in improving my manuscript. I read comments by reviewers repeatedly, purchased the recommended reference by the Reviewer 2 (“Understanding the new statistics”) and read through it, and made revisions on data analyses and presentations. Specifically, the bisection method model and GLMMs in the previous manuscript were removed together with Fig 3 and Table 1, and more simple liner mixed models have been adopted. New Fig 3 and improved Fig 2 and 4 with confidential intervals have been prepared. I also thank the editor for the editorial comments. All the flaws have been rectified in the revised manuscript.

**Reviewer #1: General Comments:**
I’ve reviewed this work multiple times. Although I think the approach is gradually progressing toward a correct statistical design, the description of the GLMM methods and results are still unclear, particularly with respect the temporal component. My best guess is that the author has used a model term of “period” (although I think they call this “Year2”, which is confusing), which has two levels. Then they have created many models, each of which splits the dataset at a different calendar year. So, in one model, “period” might have one level with 2004-2008 and the other with 2009 onwards, while in another model, one level might span 2004-2012 and the other level 2013 onwards. AICs are then used to compare all these temporal splits, along with a model with no split. I think this is what the author refers to as the “bisection method”, and seems to correspond to Figure 3, where you can see an AIC value provided for each calendar year. Although my interpretation here is somewhat contradicted by this statement - “(Variable ~ Site + Year2 + Site:Year2 + (1|Survey), REML = FALSE) where Year2 = 1*(Year > 237 2012) for fish abundance and Year2 = 1*(Year > 2011) for other variables” (Lines 236-237), which implies that the time variable has a set calendar cut-off depending on the response variable (fish abundance or other). But then how is there AIC value for each calendar year (Figure 3)? Hence my confusion.
If my above assessment is correct, it raises some concerns regarding the balance of the design, and the testing of other model terms. Regarding balance, those models that have only one or two years’ data in one level, and many in the other, are likely to have issues with power. Has this been considered? Regarding testing of other model terms, why only use AIC for the models that differ in the “Year2” model term and not those of most interest, i.e. the interaction between site and year? As far as I can tell, the importance of these model terms is assessed using p-values alone. A preferable approach would be to use AIC throughout, and simply split the “Year2” variable into “before” and “after”, based on the known year the NPP stopped operation. This would provide only two models to compare for this term, with and without “Year2”. These two models can then be compared against models that vary the inclusion of other terms, e.g. with and without the term “Site:Year2”, which is what you are really interested in. If my above assessment of the modelling approach is incorrect, the methodological description requires considerable improvement to clarify exactly what was done, how many models were used, the years they covered etc.
**Response:** The analysis using the bisection method model has been removed in the revised manuscript. This was because i) the model has a problem of balance having “only one or two years’ data in one level” as was suggested by the reviewer 1, and also ii) suspension of NPP as a treatment effect is “unreplicated” as was pointed out by the reviewer 2 so that there is not much point to conduct this analysis. Therefore, in the revised manuscript linear mixed models have been adopted separately during NPP on and off periods according to the suggestion by the reviewer 2. Providing 95% confidence intervals has made it possible to compare inter-period difference within a site and inter-site difference within a period at a glance in the new Figs 3 and 4.

**Reviewer #1: Specific Comments:**
For the analyses of species categories (tropical, temperate etc), why revert to nested ANOVAs within each site? The same GLMM approach used for total abundance, where site is a factor with three levels and an interaction term between site and year is included, seems warranted for each of these sub-categories, unless a meaningful combination response variable could be derived (e.g. “tropical proportional abundance”), which would reduce the number of separate analyses required.

**Response:** The analyses of species categories have also been revised to adopt linear mixed models to be consistent.

More detail regarding the PERMANOVA modelling is required. Was a random nested factor “year” included in the modelling? How were individual surveys within years dealt with?
**Response:** Abundance data were summed for each species in each year, rather than modelling nested PERMANOVA. This has been clarified in the revised manuscript (L 156-157).

**Reviewer #2: General Comments:**
General
More description has been provided of sample data collection from survey sites.
Questions on the multidimensional scaling analysis that is presented in Fig. 5, have been answered (but not for the manuscript).
Temperature data for the 3 marine sites has been provided in Table S1. This was very helpful for me to try mixed model analysis and presentation of findings (see attachment).
References have been provided for some of the statistical tests described in the methods section. However, could references for the multi-dimensional scaling analysis be provided?, and for the Bisection analysis? (if retained)
On line 193, should "year" be "year2"?. Should "year2" be better named throughout, to indicate time period for Otomi NPP state of operation ("on"/"off")?.
**Response:** I thank the reviewer for valuable comments. The description of the multidimensional scaling analysis has been described more in detail in the revised manuscript (L 156-158). The reference for the multi-dimensional scaling analysis (Anderson 2001) has been cited, while bisection analysis has been deleted in the revised manuscript.

**Reviewer #2: Specific Comments:**
Statistical analysis
The manuscript now seeks to directly tackle analysis of differences in response variables across the time periods when the Otomi NPP was operating and when it was not. That is, 2-factor ANOVA's were dropped, and mixed model analyses adopted instead. However, I believe further improvement to these analyses may be needed, if I correctly interpret what was done.
A thing to note is that the design for the main treatment effect -Otomi NPP operation- is unreplicated over the study. This is necessarily the case of course, given how the data could be collected, but it has implications to the analysis, because trend in response variables over years might be confounded with Otomi NPP operating period effects. The major implication is that the Otomi "on"/"off" effect can't be directly tested via a single statistical model. A way of get around this limitation is to use confidence intervals to depict differences between operating periods, and to show how sites differ when - Otomi NPP - is operating, and when it isn't. (Example figure to illustrate this is provided for temperature data; in attachment). [A useful reference to using 95% confidence intervals instead of p-values is Cumming G. (2012). Understanding The New Statistics. Effect Sizes, Confidence Intervals, and Meta Analysis. Routledge, Taylor & Francis Group.]
**Response:** I thank the reviewer for introducing Cumming (2012). I purchased the book and found it to be fascinating. I was surprised to see that none of statistical textbooks published in Japan, at least those I have checked, is referring this work. The concept described in the book seems sensible and quite revolutionary, yet I still hesitate to totally abandon the “null hypothesis significant testing” attitudes. I however have understood the usefulness of providing confidence intervals. Standard errors have been thus replaced by 95% confidence intervals in the revised manuscript. This way complications of *p*-value descriptions have been avoided in the revised manuscript. This method has been described in detail in the revised manuscript (L140-154).

The mixed model used to analyse the study's data across the (Otomi NPP) operating periods, is given line 194. This is more R-code than a direct model description, and the actual variables used are not clear, eg. what is "Survey". "Survey" is mentioned previously on line 143, but is then not clearly enough defined for a description of the statistical model. I suggest a clearer description of the model, perhaps:-
response ~ Site (fixed Effect) + Year/survey (random Effects)
Separate analyses for NPP "on", and "off". "Survey" is the replicate data nested below Years; both as random effects in the model.
I think it is reasonable to separately analyse the data for when Otomi NPP is "on", and when it is "off", given this treatment is unreplicated. This has the advantage of simpler statistical models and analyses, and avoids any interaction complications when extracting parameter estimates from analyses. Any "Interaction" effects are then simply embedded in estimated means and confidence intervals, and can be evaluated visually while comparing sites for each response variable.
Tests of difference between fixed effect levels in mixed models are produced by the lme4 software, but the F tests reported are controversial among mixed-model authorities, Chi-square tests being better regarded, and these in turn are less well regarded than direct comparison of estimated confidence intervals (example for temperature data shown below). Data+models will differ in degree of these problems, but it might be best to adopt the best accepted presentation method. The author's reference, Bates et al (2015) covers these issues.
The "mixed model" analyses used for data analysis were described in the manuscript as GLMM's, I believe they might actually be LMM's. GLMM's use non-normal error terms and non linear "link" functions between data and model, and are even more problematic to interpret than LMM's. LMM's can accomodate transformations of the response variable (ie. sqrt, log, etc), without the analyses becoming GLMM's. I believe the "G" letter should be dropped from GLMM throughout this manuscript.
**Response:** I checked references carefully and understood that linear mixed model rather than GLMM is more suitable for the present data set. Also the data analyses have been conducted separately in the NPP on and off periods in the revised manuscript as was suggested by the reviewer 2.

Bisection analyses
Perhaps partly in response to a previously suggested uncertainty about whether the Otomi NPP was operating at its previous level when the 2012 surveys were conducted, the author has included a "Bisection" analysis to test when the the Otomi (only?) data can be empirically broken into 2 uniform time periods. I may be misinterpreting the details around this in the manuscript. If retained, this content may need to be better introduced and wht it shows more clearly described. A reference for this analysis may also be useful. I didn't see any discussion of the outcome of the "Bisection" analyses, so my question is, how did these analyses improve the study's analysis and major findings?. This material may even weaken the study, if it shows changes in response variables preceeded changes to Otomi NPP being switched off. This content comes back to the question in my previous review, was the Otomi NPP being wound down well before the 2012 Otomi survey?, such that an effect could be seen in the 2012 survey at Otomi (for temperature and fish assemblages), or were the 2011/2012 effects at Otomi also partly seen at other sites, and therefore due to a more general environmental effect?. This content then should be more clearly discussed if retained in the manuscript.
If the Bisection analyses don't improve clarity toward resolving this issue of Otomi possibly being wound down prior to the 2012 survey, and details on the Otomi NPP power output (prior to the 2012 survey times) can't be accessed, should the "Bisection" analyses be retained in the manuscript?. If not, the data could just be analysed "as is" (despite the 2012 Otomi on/off uncertainty), strong effects in the study will still be demonstrated.
**Response:** After carefully considering comments by both reviewers I have deleted the bisection method analyses.

Presentation
Table 1.
Contains parameter details for site and "year2" (~NPP operating time intervals?). Some of this parameter information is also in Figure 4, so is presented in both "figures and table" (usually not acceptable), and so should be in one or the other. Table 1 does additionally contain p-value information for F and t tests of significance between fixed effect levels (ie. with site and "year2"), but these are poorly regarded (outlined above), and presents parameter estimates in a way that can be confusing to people not familiar with this statistical model format. The F and t tests could be dropped, and Chi-square tests for simply differences between "sites" could be presented instead. Parameter values for each site are then in an updated Fig. 4 (suggestion below), instead of in Table 1. Tests for differences between Otomi time periods (on/off) are not needed in models, if analyses for Otomi "on" and Otomi "off" are conducted separately (and reported separately within Table 1, with site estimates presented in Fig 4 (suggestion)). This reporting for each variable in the current Table 1, could be extended to the Tropical, Sub-tropical and Temperate species subdivisions within the Abundance and Richness variables. Tests for these subdivisions currently appear obscurely as labels in Figure 4, and introduce some clutter into the figure.

**Response:** Table 1 has also been deleted in the revised manuscript, as it was accompanied with the bisection method model analyses and GLMMs.

Figure 2.
This Figure contains un-necessary information in the form of standard errors for each year*site data point. With the dropping of the 2-factor ANOVA approach to analysis, this information is no longer really needed, and would instead be provided more directly in an updated Figure 4 (see below). However, this figure can still provide useful information, in depicting how the raw data varies over sites. For the case of the temperature data, adding lines to adjacent year means, gives a more clearly digestable idea of how sites can be quite consistently ranked for mean temperature over the series of years that were studied, and showing strongly the effect of the heat source being cut at Otomi around 2012 (and perhaps the same shown for "fish abundance", etc...).
So perhaps confidence intervals can be dropped from Fig. 2, and lines added linking adjacent year means for each site. This altered figure is especially useful for temperature, as this is driving the biological effects in the study.
**Response:** I thank the reviewer again for this constructive comment. Data presentation has been changed to provide row data plot and year means drawn as lines for each site as was suggested in the revised Fig 2.

Figure 3.
discussed above, still needed?
**Response:** The Figure has been deleted together with the bisection method model analyses.

Figure 4.
Error bars presented in Figure 4 of the previous manuscript, were changed from standard deviations to standard errors. This is an improvement but still not optimal. I have included in an attachment, some R-code on how to calculate 95% confidence intervals for fixed effects from mixed models, by "simulating" mixed model fits (from lmer(model) ), and then extracting 0.025 and 0.975 percentiles. To illustrate, this was done for the temperaure data now included in Table S1, for when Otomi NPP was operating, and when it wasn't. The confidence intervals have been plotted in an attached Figure, generated by R (code attached). I recommend using 95% CI's, because significant differences can be noted directly from these plots, eliminating the need to provide information currently in Table 1. The author might need to reconsider grouping separate plots in Fig. 4 around variables (for greatest test validity), rather than sites, as is currently the case. Temperature confidence intervals could be included in Figure 4, or a separate small similarly structured Figure.
A rule of thumb applies to presentations comparing treatment means via 95% confidence intervals. Where 2 intervals just fail to overlap, the treatment levels differ at approximately p=0.01. Confidence intervals will differ at p~0.05 if the overlap is about a third of one side of an an error bar (Cumming (2012) ).
Please note that Figure 4 should plot both upper and lower confidence intervals, to enable quick visual comparisons to be made between "sites"/"treatments". Bar plots can be used to plot estimated means, or points, as in the temperature example provided, but any bar shading should obviously be light enough not to obscure the lower bars.
The author plotted standard errors in his new Figure 4, because his prefered plotting software (Kaleida Graph) couldn't draw 95% CI's. If Kaleida Graph does not have an option to plot user supplied errors (where this can't be directly generated within the software), other plotting software to do this is available. R can be programmed to produce such plots (see attached document for code), though time consuming to implement.
**Response:** I sincerely thank the reviewer for providing the R code. I applied this procedure to each response variable, resulting in the new Figure 3 and revised Figure 4. Means and confidence intervals were obtained through the R codes with simulation, then graphs were drawn by Kaleida Graph with these data.
