# Supplementary material for: Tropical fishes vanished after the operation of a nuclear power plant was suspended in the Sea of Japan
Source: PLoS One. 2020 May 6;15(5):e0232065. doi: 10.1371/journal.pone.0232065 (PMC7202591; doi:10.1371/journal.pone.0232065)
Supplement: Attachment — Submitted filename: Response to the Editor.docx [file pone.0232065.s008.docx]

**Response to the Editor**

Line 42: Should be ‘districts’

Line 50: Delete ‘the’ before ‘extension’

Response: I have corrected the above two points according to the suggestion.

Lines 136-139: I find this text a little confusing as written. Maybe rewrite as something like:

‘The center of distribution (COD), defined as the mean southern and northern latitudinal limit of distribution in the northern hemisphere, was calculated using Nakabo [19] for all recorded species. The COD was then compared to the mean latitude recorded in each survey and used as a criteria of spatio-temporal tropicalization.’

Does that work? That seems to be what has been done but it is not entirely clear from the text as it is written.

Response: I thank the editor for notifying this ambiguity. I have made revisions on these sentences as follows (L136-139):

The center of distribution (COD), defined as the mean southern and northern latitudinal limit of distribution in the northern hemisphere, was calculated using Nakabo [19] for all recorded species. The mean of COD in each survey was then used as a criteria of spatio-temporal tropicalization.

Line 14: delete ‘of’ before ‘lme4’

Response: I corrected it according to the suggestion.

Line 145: provide the years the NPP was in operations in parentheses after ‘operation’

Line 146: Time period confusing as written. Should be simplified to something like ‘after 20 february 2012’ or something similar

Response: I have revised this part as following (L 144-146):

The analyses were conducted separately in two periods when NPP was in operation (from 2004 to early February 2012) or suspended (February 20, 2012 and later).

Line 149: What does ‘above category’ mean>

Response: I have clarified this phrase as follows (L 149-150):

above category (tropical, subtropical and temperate species)

Line 151L Write as ‘by simulating linear mixed model fits (10,000 runs)

Line 152: ‘significant’ is misspelled

Line 153: Need a comma before ‘as well’

Line 178: Write as ‘Lines represent yearly means for each site (surveys = 4)’ or something like that

Line 182: Shouldn’t this be ‘and later’?

Lines 184-185: Write as ‘Error bars represent 95% confidence intervals.’

Line 204: Should be ‘higher tropical fish abundance’

Line 208: Should be ‘Mean temporal changes’

Lines 210-211: Write as ‘Error bars represent 95% confidence intervals.’

Line 217: Delete ‘either’

Line 229: Should be ‘the NPP’ and need a comma after ‘suspension’

Line 264: write as ‘and therefore the small increase’

Line 283: Should be ‘effects’

Line 295: I assume this means that larval fish spawned in this area likely self-recruited so should be something like ‘Locally spawned larval fish of these species may have…..’

Response: Corrections have been made according to the above suggestions.
